# Transcript- and Protein-Level Preservation and Spatial Reorganization of EMT and Vascular–Mesenchymal Gene Programs (*SNAI1*, *TGFB1*, *PECAM1*, *VIM*) in Human Fetal Kidneys with Congenital Anomalies of the Kidney and Urinary Tract (CAKUT)

**DOI:** 10.3390/genes17091028

**Published:** 2026-08-28

**Authors:** Lucija Bavčević, Anita Racetin, Petar Todorović, Sandra Kostić, Sandra Zekić Tomaš, Katarina Vukojević, Nela Kelam

**Affiliations:** 1 FH Oberösterreich Campus Wels, Bio- and Environmental Technology, University of Applied Sciences Upper Austria, Roseggerstraße 15, 4600 Wels, Austria; lucija.bavcevic2@students.fh-wels.at; 2 Department of Anatomy, Histology and Embryology, University of Split School of Medicine, Šoltanska 2A, 21000 Split, Croatia; anita.racetin@mefst.hr (A.R.); petar.todorovic@mefst.hr (P.T.); katarina.vukojevic@mefst.hr (K.V.); nela.kelam@mefst.hr (N.K.); 3 Center for Translational Research in Biomedicine, University of Split School of Medicine, Šoltanska 2A, 21000 Split, Croatia; 4 Department of Pathology, Forensic Medicine and Cytology, University Hospital of Split, Spinčićeva 1, 21000 Split, Croatia; sandra.zekic-tomas@mefst.hr; 5 Department of Pathology, University of Split School of Medicine, Šoltanska 2, 21000 Split, Croatia; 6 Mediterranean Institute for Life Sciences (MedILS), University of Split, Meštrovićevo Šetalište 45, 21000 Split, Croatia

**Keywords:** CAKUT, SNAI1, TGFB1, PECAM1, VIM, epithelial–mesenchymal transition, human fetal kidney, renal dysplasia, immunofluorescence, colocalization

## Abstract

Background/Objectives: Congenital anomalies of the kidney and urinary tract (CAKUT) are a leading cause of pediatric kidney disease. Epithelial–mesenchymal transition (EMT), governed by SNAI1 and TGF-β, and a vascular–mesenchymal program marked by PECAM1 and VIM are central to nephrogenesis, but whether these programs are transcriptionally activated in human CAKUT is unknown. We assessed their expression and spatial organization in human fetal kidneys. Methods: We reanalyzed public transcriptomic datasets for six transcripts (*SNAI1*, *TGFB1–3*, *PECAM1*, *VIM*) and performed quantitative double immunofluorescence for four gene products (SNAIL, TGF-β1, CD31, vimentin) on formalin-fixed human fetal kidneys (20 controls, 19 CAKUT), with colocalization quantified by Pearson’s coefficient. Results: No statistically significant difference in transcript or protein abundance was detected between control and CAKUT kidneys, in either the cortex or the medulla, and abundance did not change across developmental phases. In contrast, spatial colocalization of SNAIL–TGF-β and of CD31–vimentin was increased in CAKUT kidneys. Conclusions: These findings may suggest that, in human fetal CAKUT, EMT and vascular–mesenchymal programs are not quantitatively upregulated but instead show altered spatial organization of otherwise unchanged gene products. As colocalization reflects spatial proximity rather than molecular interaction, these observations are correlative and warrant functional validation.

## 1. Introduction

Congenital anomalies of the kidney and urinary tract (CAKUT) encompass a heterogeneous spectrum of malformations—including renal hypoplasia, dysplasia, horseshoe kidney and ureteric anomalies—and constitute the most common genetic cause of chronic kidney disease in children [1,2]. CAKUT is genetically heterogeneous: monogenic forms arise from pathogenic variants in transcription factors and signaling genes that orchestrate nephrogenesis, including *PAX2*, *HNF1B*, *EYA1*, *SIX1*, *SALL1*, *RET*/*GDNF* and *BMP4* [3,4,5]. Yet, identified variants explain only a minority of cases, and—more fundamentally—it remains unclear how the downstream gene-regulatory programs controlled by these genes are deployed in the malformed human kidney.

Nephrogenesis is driven by reciprocal induction between the ureteric bud and the metanephric mesenchyme, and proceeds through defined phases determined by ureteric bud branching and its capacity to induce nephrons [6]. Nephron formation is itself a change in cell state: the SIX2-positive cap mesenchyme condenses around each ureteric tip and undergoes a mesenchymal-to-epithelial transition (MET) to generate the renal vesicle and, ultimately, the nephron epithelia [6,7]. The machinery that executes this conversion—the mesenchymal-to-epithelial transition (MET) program, governed by the master transcription factor *SNAI1* and driven by TGF-β signaling (*TGFB1*–*TGFB3*)—must therefore be held under tight spatial and temporal control during development [8,9,10]. This control is directional: *SNAI1* is downregulated as mesenchymal cells epithelialize, in part through its direct repression of the differentiation factor *HNF1B*—itself a CAKUT gene—and its aberrant reactivation in the mature kidney is sufficient to drive EMT and fibrosis [11,12]. What matters, then, is not simply whether these programs are present, but where and when they are engaged. In parallel, a vascular–mesenchymal program, marked by the endothelial gene *PECAM1* and the mesenchymal intermediate-filament gene *VIM*, patterns the developing renal vasculature and interstitium [13,14]; endothelial and mesenchymal states in this compartment are themselves interconvertible through endothelial-to-mesenchymal transition (EndMT), a recognized source of interstitial cells in renal injury [15].

In acquired renal injury—most clearly in unilateral ureteral obstruction—these programs are unequivocally activated at the transcriptional level [12,16]. Whether the same is true of congenital malformation is far less clear, and the assumption that CAKUT involves transcriptional upregulation of EMT and vascular–mesenchymal genes has not been directly tested in human fetal tissue. Crucially, gene expression can be disrupted in two distinct ways: a program may be quantitatively up- or downregulated, or it may be expressed at normal levels but deployed incorrectly in space, with its products distributed and juxtaposed abnormally within the tissue. Standard readouts, whether differential expression analysis or quantification of immunofluorescence signal intensity, capture only the first possibility. The second requires a measure of how gene products are arranged relative to one another, which colocalization analysis provides but is rarely applied in this setting.

We therefore combined transcript-level and protein-level analysis of six transcripts (four gene products) in CAKUT. First, we asked whether *SNAI1*, *TGFB1–3*, *PECAM1*, and *VIM* are differentially expressed in human CAKUT tissue using publicly available transcriptomic data and, as an external acquired-injury contrast for inducibility, a murine model of acquired obstructive injury. Second, we mapped the corresponding gene products in human fetal kidney by double immunofluorescence and asked whether their abundance is altered, taking the developmental stage into account. Third, and independently of abundance, we asked whether the spatial co-organization of the EMT pair (SNAIL–TGF-β1) and the vascular–mesenchymal pair (CD31–vimentin) is altered, and whether any such reorganization is confined to kidneys in which tissue architecture is preserved or extends across the CAKUT spectrum, including dysplasia.

## 2. Materials and Methods

### 2.1. Differential Gene-Expression Analysis of Publicly Available Datasets

Transcriptomic datasets were retrieved from the NCBI Gene Expression Omnibus (https://www.ncbi.nlm.nih.gov/geo/; accessed on 12 June 2026). GSE83946 comprises ureteric tissue from human CAKUT cases and controls (7 controls, 19 CAKUT) and was used to test whether the genes of interest are differentially expressed in human congenital malformations [17]. GSE42303 comprises murine kidney tissue following unilateral ureteral obstruction (9 sham, 9 UUO) and was included as an external acquired-injury contrast for the inducibility of the same programs [18]. GSE42303 has a factorial design (wild-type, *TIMP2*-/- and *TIMP3*-/-; sham and UUO). To avoid a genotype confound, the primary analysis was restricted to wild-type animals (3 sham vs. 3 UUO).

Series matrices were obtained from GEO; intensities were log2-transformed where necessary and quantile-normalized across arrays. Genes detected above background in only a minority of samples were flagged and not interpreted. Where several probes mapped to one gene, the probe with the highest mean expression was used. Differential expression of *SNAI1*/*Snai1*, *TGFB1–3*/*Tgfb1–3*, *PECAM1*/*Pecam1*, and *VIM*/*Vim* was assessed in limma [19] with an empirical-Bayes-moderated linear model (design~group) and Benjamini–Hochberg FDR control [20]; the two datasets were analyzed separately, so no cross-dataset batch correction was applied. Analysis code and specimen-level values are provided (Supplementary Materials).

### 2.2. Tissue Specimens and Ethics

Human fetal kidney specimens were obtained from the Departments of Gynaecology and Obstetrics and of Pathology, University Hospital of Split, following spontaneous abortions or tubal (ectopic) pregnancies, and archived at the Department of Anatomy, Histology and Embryology, University of Split School of Medicine. The cohort comprised 20 control kidneys without renal malformation and 19 CAKUT kidneys: duplex/ureteric anomaly (*n* = 2), renal hypoplasia (*n* = 3), horseshoe kidney (*n* = 4) and renal dysplasia (*n* = 10). Developmental (postconceptional) age was determined from menstrual data, crown–rump length, and the Carnegie staging system and, for phase-stratified analyses, assigned to Ph2 (15–22 weeks), Ph3 (23–35 weeks), and Ph4 (≥36 weeks). One control specimen (14 weeks) fell into Ph1 (<15 weeks); as Ph1 was represented by a single specimen, it was excluded from phase-stratified comparisons. A further control specimen lacked a recorded developmental age and was excluded from phase-stratified and age comparisons but retained in the overall control-versus-CAKUT abundance analysis. Sex was not recorded for these archival specimens. The exact diagnosis and associated anomalies of each specimen are listed in Table S1. Developmental age differed between groups (control median 21.5 weeks vs. CAKUT 30.5 weeks; Mann–Whitney U, *p* = 0.004).

The study was conducted according to the guidelines of the Declaration of Helsinki. Ethical approval was granted by the ethics committee of University Hospital Centre Split (class: 500-03/23-01/187, protocol code no. 2181-147-01-06/LJ.Z.-23-02, date of approval: 20 September 2023) and the ethics committee of the University of Split, School of Medicine (class: 003-08/23-03/0015, protocol code no. 2181-198-03-04-23-0073, date of approval: 27 September 2023).

### 2.3. In Situ Mapping of Gene Products by Double Immunofluorescence

Tissue was fixed in 4% paraformaldehyde in 0.1 M phosphate-buffered saline (PBS), dehydrated through a graded ethanol series, embedded in paraffin, and serially sectioned at 5 µm. Sections were mounted on glass slides, deparaffinized in xylene, and rehydrated stepwise in ethanol. Heat-induced epitope retrieval was performed in 0.01 M citrate buffer (pH 6.0) at 95 °C for 30 min in a water steamer. After equilibration to room temperature, slides were rinsed in 0.1 M PBS and pretreated for 20 min with a protein blocking reagent (ab64226, Abcam, Cambridge, UK) to suppress nonspecific binding. Primary antibody pairs—SNAIL with TGF-β1, and CD31 with vimentin—were applied and incubated overnight in a humidity-controlled chamber (Table 1). The TGF-β antibody detects TGF-β1; no protein-level inference is made for TGF-β2 or TGF-β3. Slides were washed in PBS and incubated with the corresponding fluorophore-conjugated secondary antibodies for 1 h at room temperature (Table 1). Nuclei were counterstained with 4′,6-diamidino-2-phenylindole (DAPI) after a final PBS wash, and sections were mounted in Immuno-Mount medium (Thermo Shandon, Pittsburgh, PA, USA). Antibody specificity was confirmed by preadsorption controls, in which each primary antibody was preincubated with the corresponding peptide antigen; under these conditions, no immunoreactivity was detected. Omission of the primary antibodies yielded no detectable signal.

### 2.4. Imaging and Quantification of Gene-Product Abundance

Immunofluorescence imaging was performed on a BX51 fluorescence microscope (Olympus, Tokyo, Japan) equipped with a DS-Ri2 digital camera (Nikon Corporation, Tokyo, Japan) and NIS-Elements F software (version 3.0). For each specimen, ten non-overlapping, systematically sampled fields were captured per compartment at ×40 magnification, with constant gain and exposure settings across all samples. Fields were acquired separately from the cortex and from the medulla. In dysplastic kidneys, which lack a morphologically identifiable corticomedullary boundary, fields were sampled across the whole tissue. Representative hematoxylin–eosin-stained sections were imaged in brightfield (transmitted-light) mode on the same microscope.

Quantification was performed in ImageJ (version 1.54g; NIH, Bethesda, MD, USA) by an operator blinded to group [21]. The proportion of each field occupied by positive signal (area percentage) was determined after median-filter background subtraction and intensity-based thresholding, and corresponded to the fraction of pixels above a defined intensity threshold relative to the total number of pixels in the analyzed region, as described in our earlier studies of the human fetal kidney [22,23,24]. Background thresholds were set on negative-control images by experienced histologists. Per-specimen values were expressed as the mean across the fields of a given compartment; for dysplastic kidneys, all fields were averaged into a single whole-tissue value. In a secondary analysis, cortical and medullary values were combined into a field-weighted whole-specimen mean. Inter-rater agreement, assessed on a random subset by two independent assessors using the intraclass correlation coefficient, was excellent (ICC = 0.86) [25]. For colocalization, fields in which Costes automatic thresholding failed to converge were excluded; a sensitivity analysis including all acquired fields yielded concordant results.

### 2.5. Colocalization Analysis of Gene Products

Pixel-level colocalization between paired gene products was quantified using the JACoP plugin [26] in ImageJ v1.54g. For each field, the green and red channels were split, the background was subtracted (rolling-ball, radius 50 px), and Costes’ automatic thresholding was applied independently to each channel [27]. Pearson’s correlation coefficient, Spearman’s rank coefficient, Manders’ coefficients M1 and M2, and the overlap coefficient were computed. Costes’ randomization (200 iterations) confirmed that the observed colocalization significantly exceeded that expected from a random pixel distribution. Fields in which automatic thresholding failed (threshold value > 30, indicating collapse of the thresholding routine) were excluded, and per-specimen values were recalculated as the mean of the remaining valid fields. Pearson’s coefficient was pre-specified as the primary metric because it is independent of thresholding; Manders’ coefficients and the overlap coefficient are reported as secondary metrics.

### 2.6. Statistical Analysis

The specimen was treated as the unit of analysis, so that each data point represents an independent biological replicate. Normality was assessed with the Shapiro–Wilk test, and tests were selected accordingly: where all groups satisfied normality, parametric tests were applied (Welch’s unpaired t-test, two-tailed; ordinary one-way ANOVA with Tukey’s post hoc test); where one or more groups departed from normality, non-parametric tests were used (Mann–Whitney U test, two-tailed; Kruskal–Wallis test with Dunn’s post hoc test). The Benjamini–Hochberg procedure was applied across the six primary colocalization comparisons (cortex, medulla and dysplasia, for each gene-product pair); both nominal *p* and adjusted q values are reported. Group × compartment effects were tested with a linear mixed-effects model (Pearson’s coefficient~group × compartment) with specimen as a random effect, accounting for paired cortical and medullary measurements; the interaction term tested whether the group effect differed between compartments. Developmental dynamics in control kidneys were modeled by linear and quadratic (second-order polynomial) regression on developmental phase, with the two models compared by the corrected Akaike information criterion (AICc). Data are presented as scatter plots showing every specimen, with median and interquartile range. Significance was set at *p* < 0.05. Analyses were performed in GraphPad Prism 10.6.1 (GraphPad Software, Boston, MA, USA) and R (version 4.3.2); figure panels were assembled in Adobe Photoshop v21.0.2 (Adobe Systems, San Jose, CA, USA). All underlying numerical values, the specific statistical test applied to each comparison, the corresponding test statistics, and both nominal and Benjamini–Hochberg-adjusted *p*-values are provided in full in Supplementary Tables S2–S9.

## 3. Results

### 3.1. EMT and Vascular–Mesenchymal Genes Are Not Transcriptionally Activated in Human CAKUT

**Figure 1:**
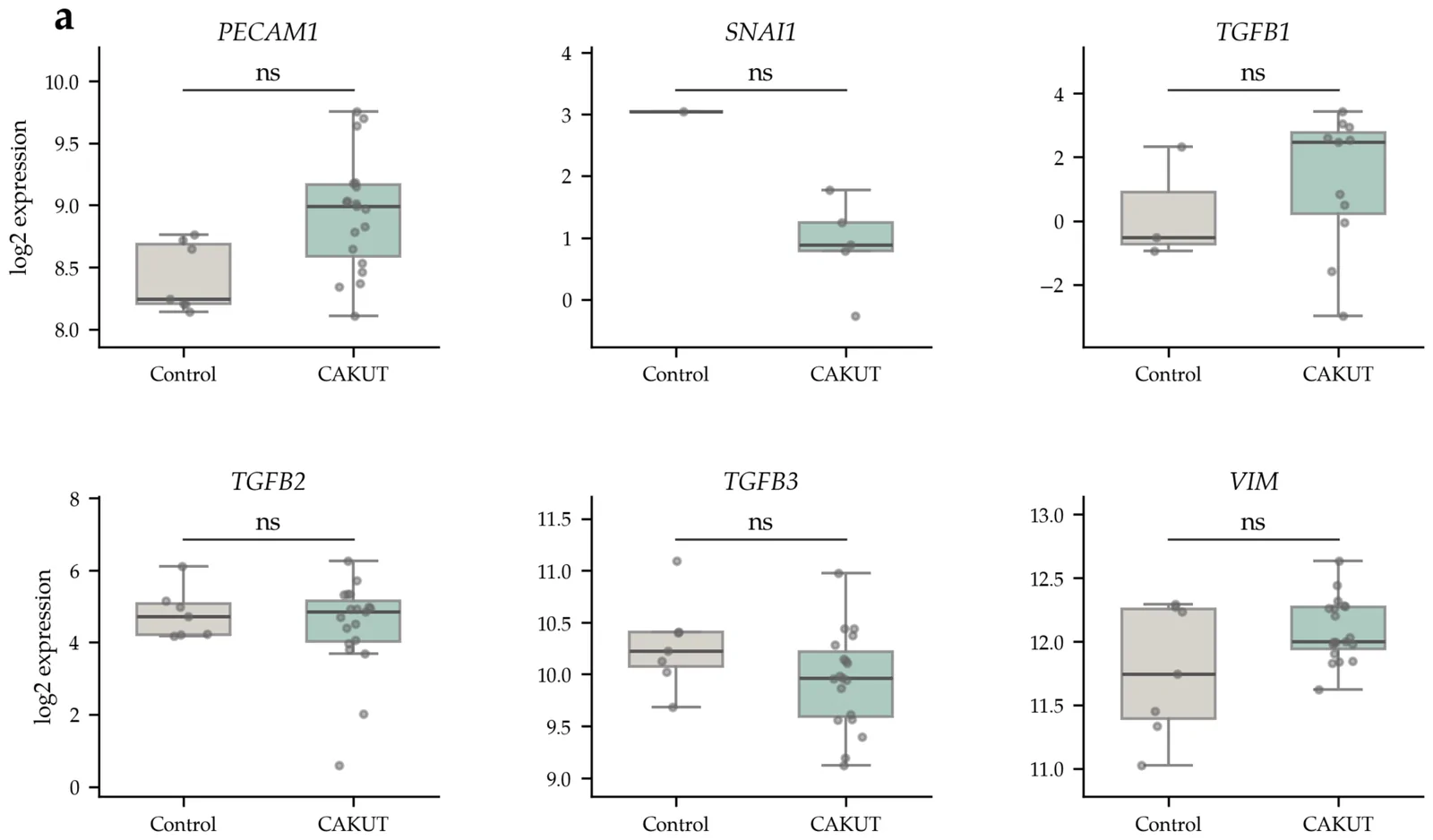

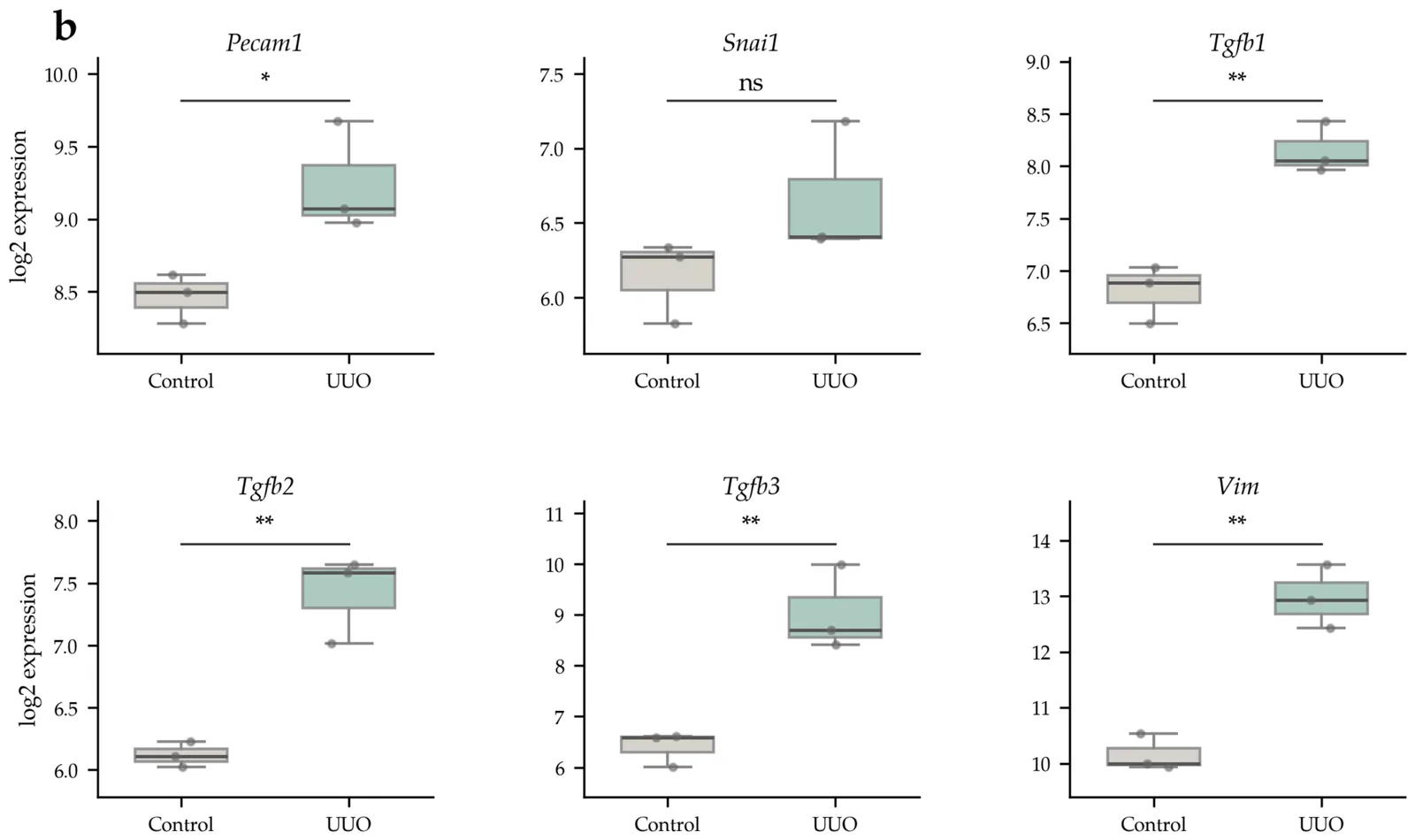
Expression of EMT and vascular–mesenchymal genes in human CAKUT and murine obstructive injury. Box plots show log2 expression of *SNAI1*, *TGFB1–3*, *PECAM1* and *VIM* in (**a**) human CAKUT ureteric tissue (GSE83946; 7 control vs. 19 CAKUT) and (**b**) murine unilateral ureteral obstruction (GSE42303; wild-type only; 3 sham vs. 3 UUO). Boxes show the median and interquartile range, whiskers show the range, and points represent individual samples. In human CAKUT, no gene survived FDR correction, and *SNAI1* was below the detection limit; in the murine model *Tgfb1–3*, *Pecam1* and *Vim* were significantly induced (FDR < 0.05), whereas *Snai1* was unchanged. Significance (Benjamini–Hochberg FDR): * FDR < 0.05, ** FDR < 0.01, ns not significant.

Differential expression analysis of human CAKUT ureteric tissue (GSE83946) showed that none of the six transcripts reached significance after FDR correction. *PECAM1* (log2FC +0.52; *p* = 0.012; FDR = 0.245) and *VIM* (log2FC +0.36; *p* = 0.028; FDR = 0.330) were nominally increased but did not survive correction for multiple testing, and the *TGFB* genes were unchanged (*TGFB1*, *p* = 0.456; *TGFB2*, *p* = 0.485; *TGFB3*, *p* = 0.133). *SNAI1* was detected in only a minority of samples (14% of controls, 26% of cases) and was close to the detection limit; its apparent decrease is therefore not interpretable (Figure 1; Table S6).

To establish that these programs are detectable and inducible using the same analytical approach, we analyzed a murine model of acquired obstructive injury (GSE42303). Here, the same genes were significantly induced in wild-type animals: *Vim* (log2FC +2.82; FDR = 0.003), *Tgfb3* (+2.63; FDR = 0.009), *Tgfb2* (+1.30; FDR = 0.007), *Tgfb1* (+1.34; FDR = 0.007) and *Pecam1* (+0.78; FDR = 0.043), while *Snai1* was not significant (FDR = 0.19) (Table S6). The EMT and vascular–mesenchymal transcriptional programs are therefore readily inducible in renal tissue but are not activated at the transcript level in human congenital malformation.

### 3.2. Gene-Product Abundance Showed No Statistically Significant Difference in CAKUT

**Figure 2:**
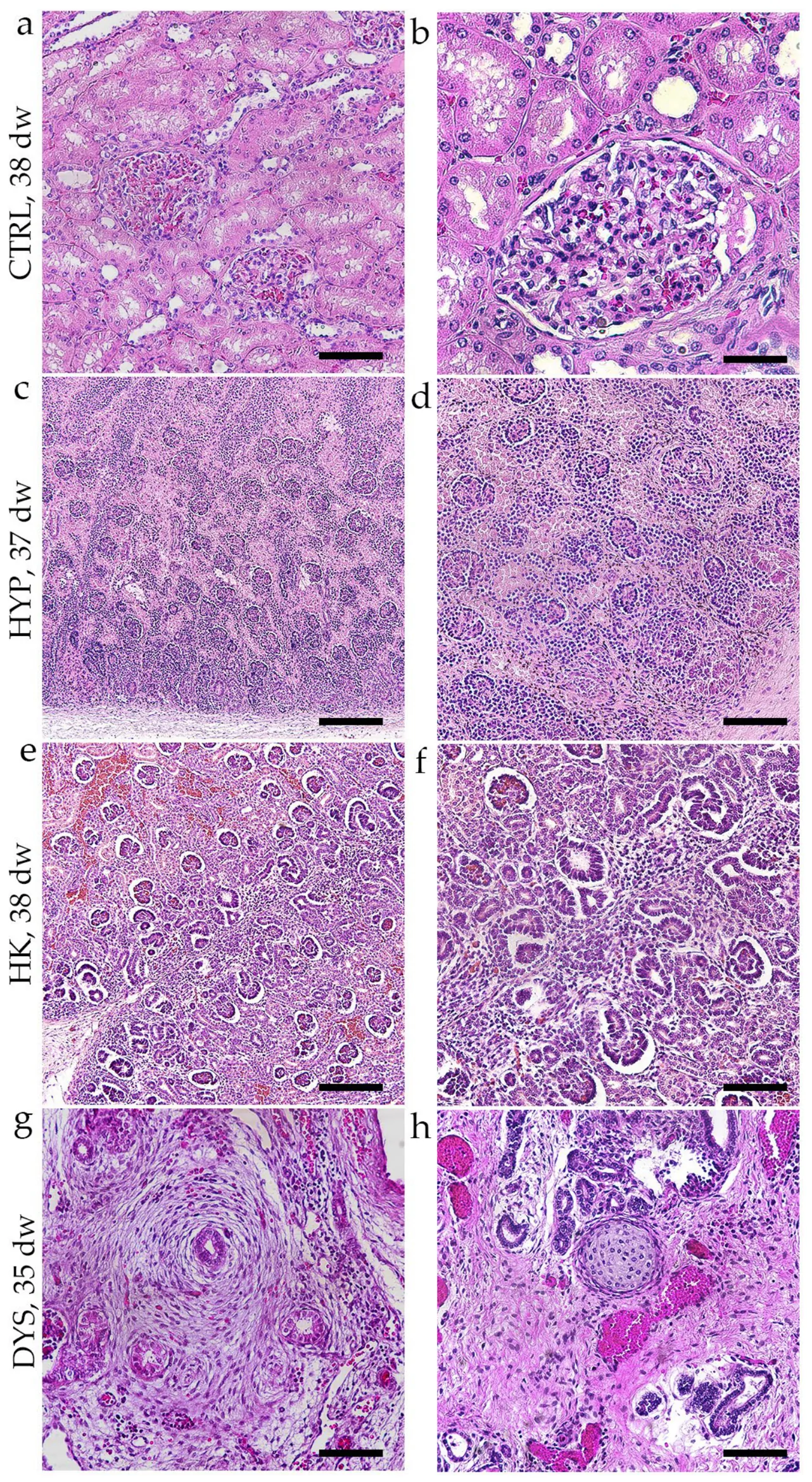
Histological characterization of the healthy kidney (**a**,**b**) and CAKUT phenotypes (**c**–**h**). Representative haematoxylin–eosin staining of a control kidney (CTRL, 38 dw), renal hypoplasia (HYP, 37 dw), horseshoe kidney (HK, 38 dw) and renal dysplasia (DYS, 35 dw), at lower (top) and higher (bottom) magnification; control, hypoplastic and horseshoe kidneys preserved the corticomedullary organization with maturing glomeruli and tubules, whereas dysplasia showed disorganized parenchyma with primitive ducts surrounded by fibromuscular collarettes and loss of the corticomedullary distinction. Images were taken at magnifications of ×100 (**c**,**e**), ×200 (**a**,**d**,**f**,**g**,**h**), and ×400 (**b**). Scale bars: 200 µm (×100), 100 µm (×200), 50 µm (×400).

Histological examination confirmed the diagnostic features of each phenotype (Figure 2). Control kidneys showed the normal architecture of the developing kidney, with a nephrogenic cortex containing maturing glomeruli and tubules overlying an organized medulla (Figure 2a,b); hypoplastic (Figure 2c,d) and horseshoe kidneys (Figure 2e,f) retained this corticomedullary organization, whereas dysplastic kidneys (Figure 2g,h) showed disorganized parenchyma with primitive ducts surrounded by concentric fibromuscular collarettes, occasional cysts and loss of the normal corticomedullary distinction. Among the phenotypes examined, dysplasia was therefore distinguished by disruption of tissue architecture, a feature that would prove relevant to the spatial analysis below.

**Figure 3:**
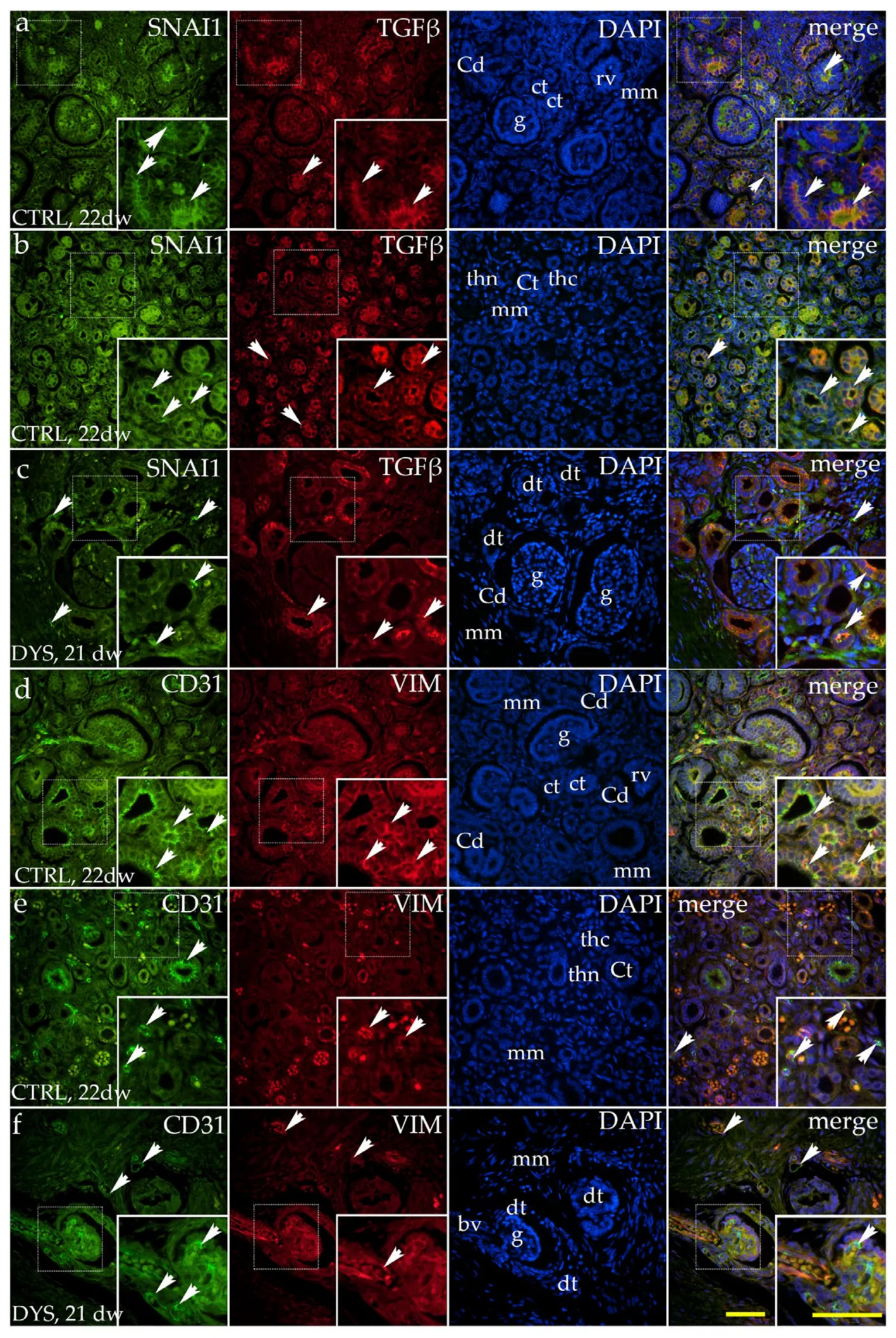
Double immunofluorescence localization of the two gene-product pairs in control and dysplastic human fetal kidney. Panels (**a**–**c**) show SNAIL (green) with TGF-β1 (red), and panels (**d**–**f**) show CD31 (green) with vimentin (red). Nuclei are counterstained with DAPI (blue), and for each field, the individual channels are shown together with the merged image. For each pair, (**a**,**d**) show the cortex and (**b**,**e**) the medulla of a representative control kidney (22 developmental weeks), while (**c**,**f**) show a representative dysplastic kidney (21 developmental weeks) imaged as whole tissue, since dysplasia lacks a corticomedullary boundary. Images were acquired at ×40 magnification under constant gain and exposure. In the merged images, colocalization of the two markers appears yellow. Arrowheads indicate representative double-positive cells; dashed boxes denote the regions shown at higher magnification in the insets. Representative cases are shown, as staining patterns were consistent across the CAKUT phenotypes examined; the images are qualitative, and the corresponding quantification is given in shown in the subsequent figures Renal vesicle (rv), metanephric mesenchyme (mm), collecting duct (Cd), glomeruli (g), convoluted tubules (ct), proximal convoluted tubules (pct), distal convoluted tubules (dct), collecting tubules (Ct), thick (thc) and thin (thn) segments of the loop of Henle. Scale bar, 50 µm (yellow lines).

**Figure 4:**
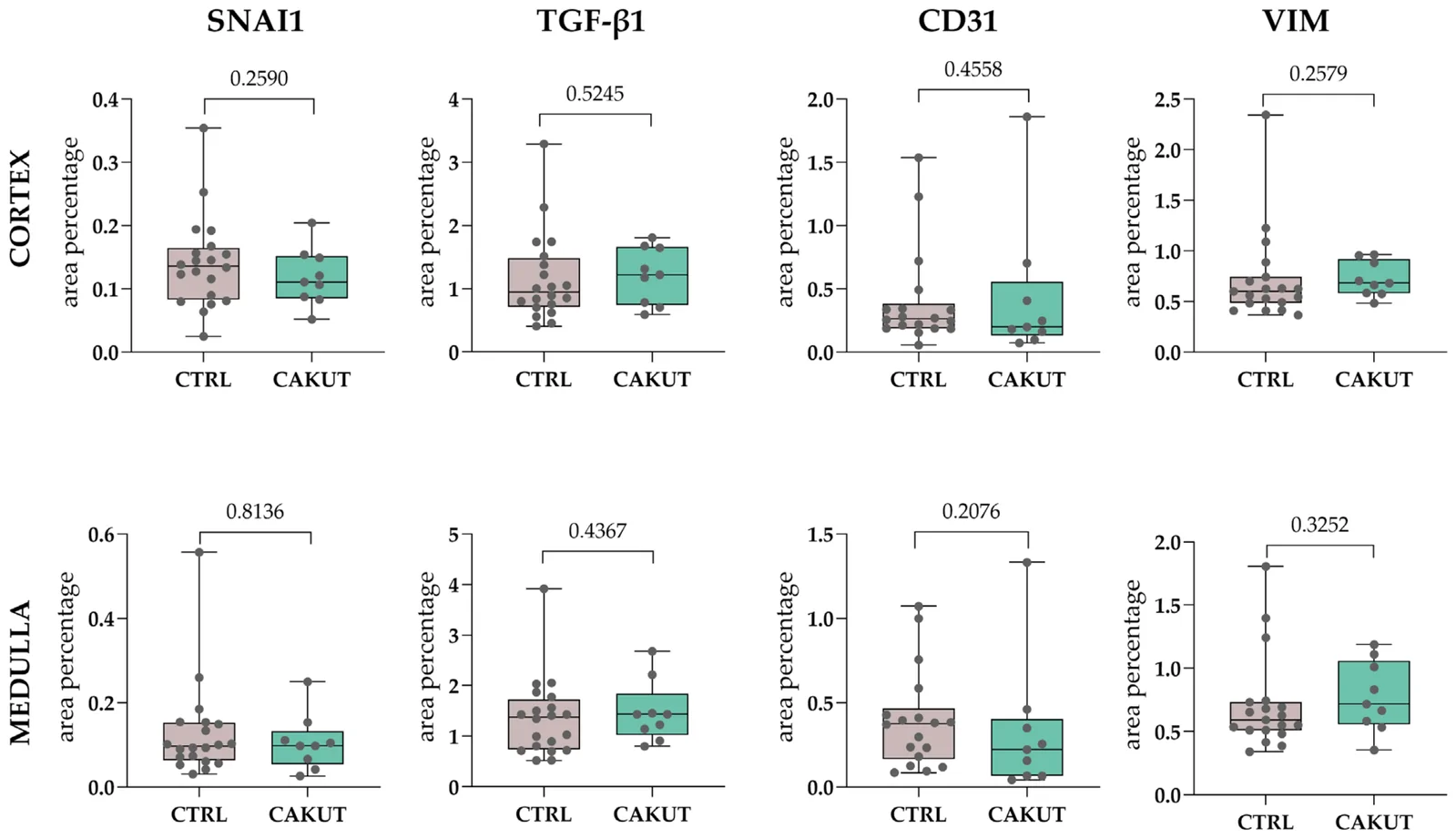
Gene-product abundance showed no statistically significant difference in CAKUT. Percentage of signal area for SNAIL, TGF-β, CD31 and vimentin in control and CAKUT kidneys, shown separately for cortex (upper row) and medulla (lower row). Each point represents one specimen; horizontal lines denote the median with interquartile range. *p*-values are from Welch’s t-test (SNAIL, cortex) or Mann–Whitney U test (all others).

To localize the corresponding gene products within this tissue, the two antibody pairs, SNAIL with TGF-β1 and CD31 with vimentin, were mapped by double immunofluorescence and imaged separately in the cortex and medulla (Figure 3a–f). Consistent with the transcriptomic data, the four gene products were readily detected in both control (Figure 3a,b,d,e) and CAKUT kidneys (Figure 3c,f). SNAIL appeared as a granular, predominantly cytoplasmic signal with variable nuclear localization in tubular epithelial and interstitial cells; TGF-β appeared as a diffuse-to-granular cytoplasmic signal in the same tubulointerstitial compartment; CD31 delineated the endothelium of glomerular and peritubular capillaries; and vimentin labeled the interstitial and perivascular mesenchyme, together with the cap mesenchyme of the nephrogenic zone in the cortex. In control kidneys, the four markers were distributed according to the normal corticomedullary organization: CD31 and vimentin were most prominent in the vascularized interstitium, and SNAIL and TGF-β1 followed the maturing tubular compartment, whereas in dysplastic kidneys the same markers instead traced the disorganized parenchyma, outlining the primitive ducts and their surrounding fibromuscular collarettes. Across control and CAKUT tissue, the intensity and extent of staining were broadly comparable, in keeping with the quantitative finding that gene-product abundance showed no statistically significant difference (Figure 4). In the merged images, each marker pair showed regions of spatial overlap (yellow); the degree of this overlap, rather than the amount of either marker, is quantified by the colocalization analysis below. A single representative case is shown per pair and compartment, as staining patterns were consistent across the CAKUT phenotypes examined.

Consistent with the transcript data, the abundance of the corresponding proteins did not differ between control and CAKUT kidneys in either compartment. In the cortex, no marker reached significance (SNAIL, *p* = 0.259, Welch’s t-test—the only marker satisfying normality; TGF-β1, *p* = 0.524; CD31, *p* = 0.456; vimentin, *p* = 0.258; Mann–Whitney U test), and the same was true for the medulla (SNAIL, *p* = 0.814; TGF-β1, *p* = 0.437; CD31, *p* = 0.208; vimentin, *p* = 0.325; Mann–Whitney U test; Figure 4; Table S2).

Abundance was also compared across the five phenotype groups (control, ureteric anomaly, hypoplasia, horseshoe kidney, and dysplasia), using a single field-weighted value per specimen (all imaged fields combined) to avoid pseudoreplication. No marker differed significantly (Kruskal–Wallis: SNAIL, *p* = 0.177; TGF-β1, *p* = 0.174; CD31, *p* = 0.713; vimentin, *p* = 0.668; Table S5).

### 3.3. Gene-Product Abundance Is Stable Throughout Nephrogenesis

**Figure 5:**
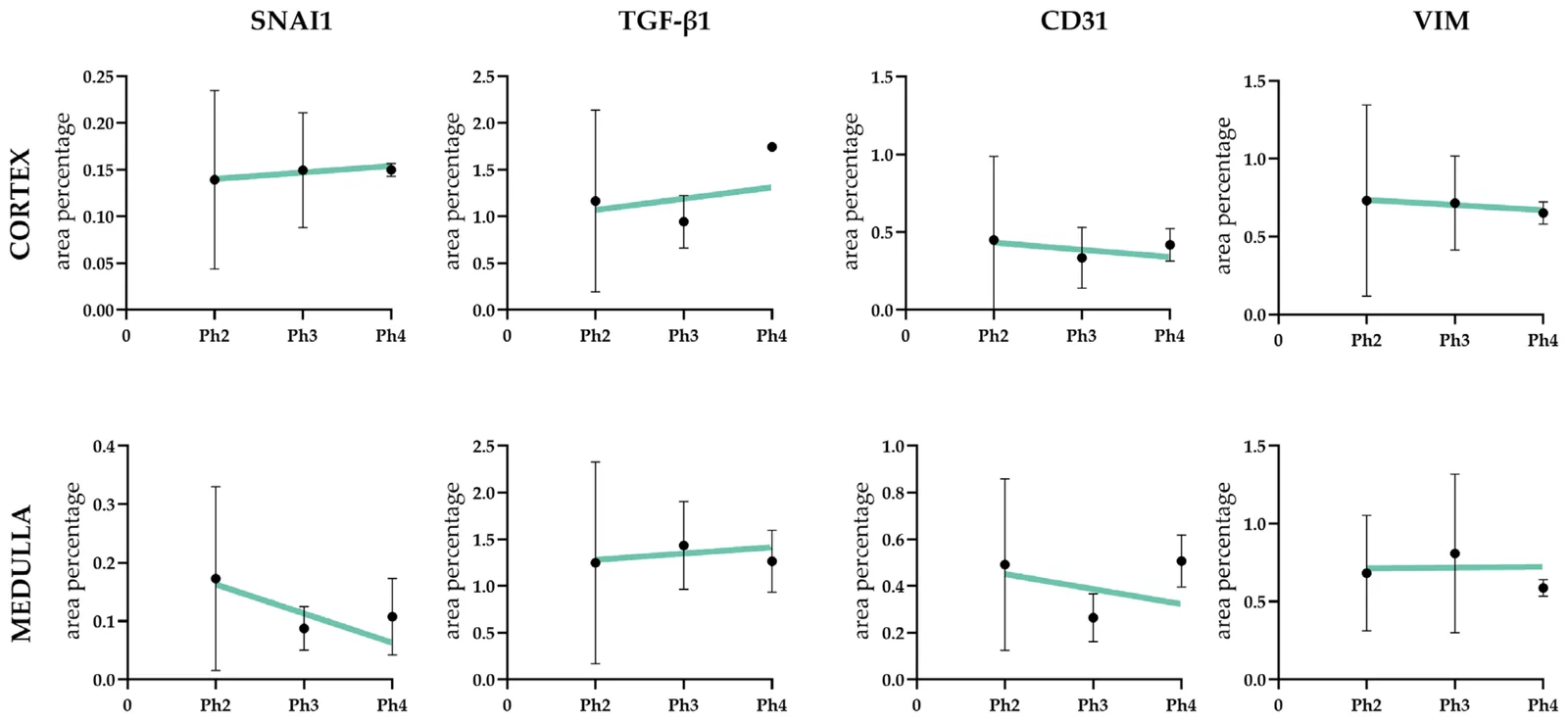
Gene-product abundance across developmental phases in control kidneys. Percentage of signal area for each marker across developmental phases Ph2 (15–22 weeks), Ph3 (23–35 weeks), and Ph4 (≥36 weeks) in the cortex and medulla. Data are presented as mean ± SD; lines connect group means.

To exclude developmental stage as a confounder, control kidneys were stratified by developmental phase: Ph2 (*n* = 9), Ph3 (*n* = 6–7) and Ph4 (*n* = 2). No marker differed significantly across phases in either compartment (Kruskal–Wallis, all *p* > 0.13; Figure 5). Modeling abundance as a function of developmental phase confirmed this: neither a linear nor a quadratic (second-order polynomial) model detected a significant trend for any marker (linear-term *p* > 0.6, R^2^ ≤ 0.02), and the simpler linear model was preferred by AICc in every case (Table S5).

Expression thus showed no significant change across the phases sampled here, so any difference observed in CAKUT cannot be attributed to differences in developmental timing.

### 3.4. Spatial Deployment of the Gene Products Is Altered in CAKUT

Colocalization was quantified for two functionally coherent pairs of gene products: SNAIL–TGF1-β1 (EMT program) and CD31–vimentin (vascular–mesenchymal program). Costes’ randomization confirmed that the measured colocalization was non-random in every specimen, validating the comparison of its magnitude between groups.

**Figure 6:**
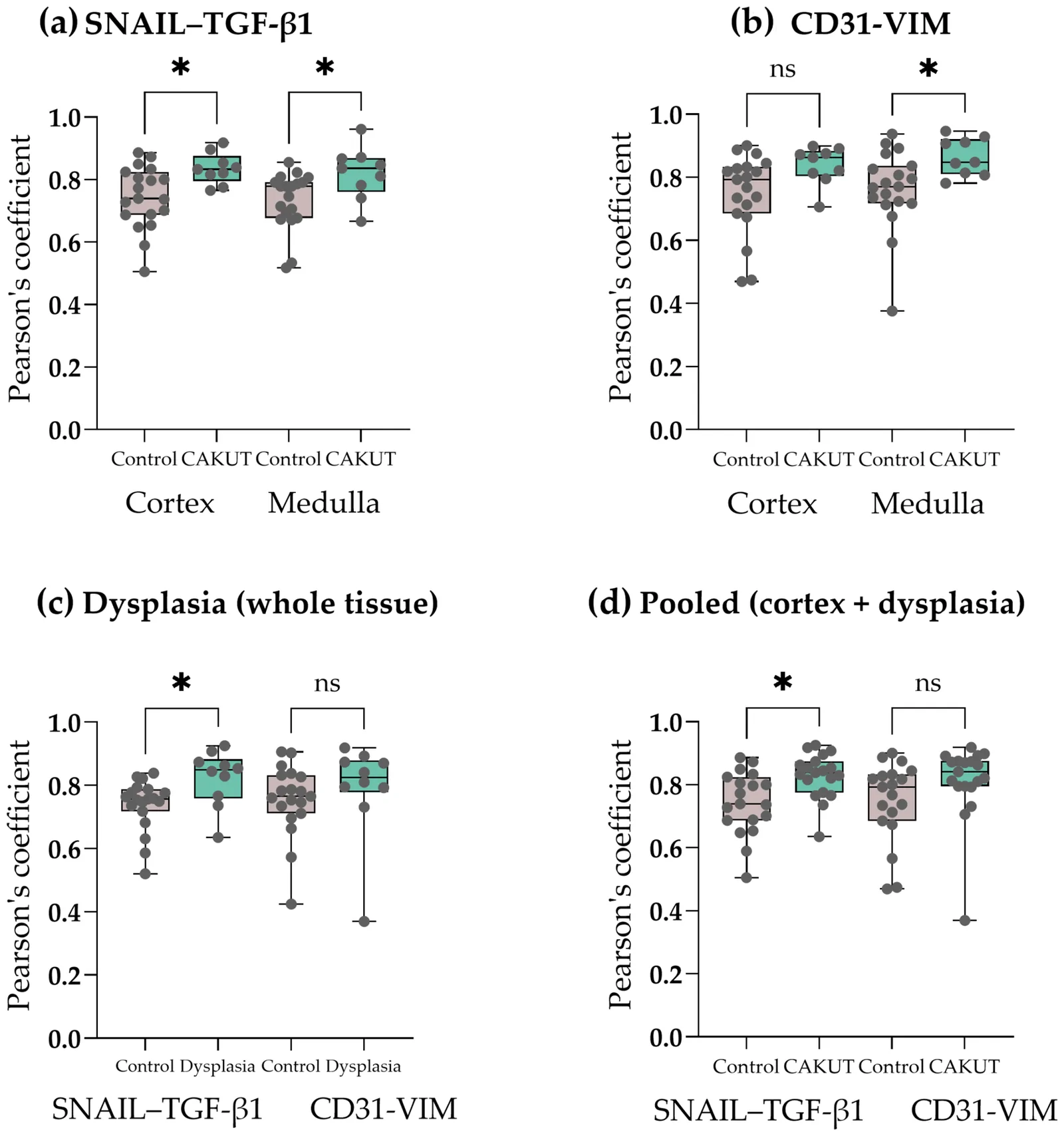
Spatial deployment of the gene products is altered in CAKUT. (**a**) SNAIL–TGF-β1 and (**b**) CD31–vimentin colocalization (Pearson’s coefficient) in control and CAKUT kidneys, cortex and medulla. (**c**) Colocalization in dysplastic kidneys, analyzed as whole tissue, compared with whole-specimen control values: SNAIL–TGF-β1 is increased (*p* = 0.008), whereas CD31–vimentin is not (*p* = 0.113). (**d**) Pooled analysis (post hoc, exploratory), combining cortical values (architecturally preserved cases) with whole-tissue values (dysplasia) (*n =* 19 per group), confirming increased SNAIL–TGF-β1 colocalization (*p* = 0.006). Each point represents one specimen; horizontal lines denote the median with interquartile range. * *p* < 0.05, ns not significant, Mann–Whitney U test; Benjamini–Hochberg-adjusted q values are given in Table S2.

In contrast to abundance, colocalization differed between groups, and the two pairs behaved differently. SNAIL–TGF-β1 colocalization was increased in CAKUT in both compartments: in the cortex (median Pearson’s r 0.83 vs. 0.74 in controls; *p* = 0.016) and in the medulla (0.84 vs. 0.78; *p* = 0.024). CD31–vimentin colocalization, by contrast, was increased overall, most evident in the medulla (0.85 vs. 0.77; *p* = 0.011), while the cortex showed the same direction of change without reaching significance (0.86 vs. 0.79; *p* = 0.062; Figure 6a,b). A formal group × compartment interaction was not significant for CD31–vimentin (mixed-effects model: +0.015, 95% CI −0.018 to +0.048, *p* = 0.38) or SNAIL–TGF-β1 (−0.005, 95% CI −0.030 to +0.020, *p* = 0.68). Although CD31–vimentin reached significance only in the medulla in the compartment-wise tests, the compartment difference was not statistically supported; therefore, it is described as an overall increase in CAKUT (group main effect *p* = 0.048) detected in the medulla. The overlap coefficient reproduced this pattern, whereas Manders’ coefficients were less informative because automatic thresholding failed in a subset of fields, which were excluded (Table S4).

Dysplastic kidneys, the largest CAKUT subgroup, lack corticomedullary architecture and were therefore analyzed as whole tissue and compared with whole-specimen control values. Here, the two pairs diverged. SNAIL–TGF-β1 colocalization was increased in dysplasia to the same degree as in the architecturally preserved phenotypes (0.85 vs. 0.76; *p* = 0.008; Figure 6c), whereas CD31–vimentin colocalization did not differ (0.83 vs. 0.77; *p* = 0.113). Tighter SNAIL–TGF-β1 coupling is therefore a feature of the CAKUT spectrum as a whole, including kidneys in which tissue architecture is lost, whereas the CD31–vimentin shift is restricted to the medulla of kidneys in which the corticomedullary axis is preserved.

Because the SNAIL–TGF-β1 shift was present in every CAKUT subgroup, a pooled analysis was performed, comparing control cortex with all CAKUT cortical measurements, together with whole-tissue values from dysplastic kidneys (*n =* 19 per group). This confirmed and strengthened the finding (0.84 vs. 0.74; *p* = 0.006; Figure 6d), whereas CD31–vimentin showed only a trend (*p* = 0.066), consistent with its restriction to the medulla. Across the six primary comparisons, four remained significant after Benjamini–Hochberg correction: SNAIL–TGF-β1 in dysplasia (q = 0.032), cortex (q = 0.032) and medulla (q = 0.036), and CD31–vimentin in the medulla (q = 0.032); CD31–vimentin in the cortex (q = 0.074) and in dysplasia (q = 0.113) did not.

## 4. Discussion

The central observation of this study is a dissociation between gene-expression level and gene-product deployment. In human fetal kidneys with CAKUT, *SNAI1*, *TGFB1–3*, *PECAM1,* and *VIM* showed no statistically significant difference from control—at the transcript level in an independent human dataset, and at the protein level across compartments, phenotypes, and developmental stages. What differs is not how much of these gene products is present, but how they are arranged relative to one another within the tissue.

This dissociation is informative rather than merely negative. EMT and endothelial–mesenchymal cross-talk are frequently invoked in renal malformation on the assumption that the corresponding genes are upregulated [8,12]; our data do not support that assumption for human congenital disease. Importantly, the murine obstruction model analyzed with the identical pipeline shows that these same genes are strongly inducible—Vim by more than seven-fold—which argues that the negative human result reflects biology rather than insensitivity of the assay or the genes selected. Congenital malformation and acquired injury therefore engage these programs differently: the malformed fetal kidney does not show the transcriptional activation that characterizes the obstructed adult kidney.

What the malformed kidney does show is altered spatial deployment, and the two programs behave differently in ways that are themselves informative. Increased SNAIL–TGF-β1 colocalization was detected in every CAKUT subgroup: in the cortex and medulla of architecturally preserved kidneys and, to the same degree, in dysplastic kidneys in which corticomedullary organization is lost. Tighter coupling of the EMT program therefore appears to be a general feature of the malformed fetal kidney, independent of phenotype and of the degree to which tissue architecture is retained. Increased CD31–vimentin colocalization, by contrast, was most evident in the medulla, indicating a more localized change in vascular–mesenchymal organization in the compartment where interstitial and vascular remodeling predominates. The increased spatial coupling of SNAIL and TGF-β1 is consistent with the well-characterized SNAIL1–SMAD3/4 transcriptional module through which TGF-β1 signaling and SNAIL cooperate, and may reflect a tightening of this axis at the protein level even where transcript abundance is unchanged [28,29]. At the resolution used here, increased SNAIL–TGF-β1 co-occurrence cannot address molecular complex formation; it is best interpreted as greater regional co-distribution of the two proteins, compatible with—but not demonstrating—co-engagement of this axis.

The compartment pattern is itself biologically coherent. In the developing kidney, the cortex houses the nephrogenic zone, in which induction, MET and active nephron assembly proceed, whereas the medulla is the site of interstitial and vascular maturation and remodeling [6]. A SNAIL–TGF-β1 module that is tighter across both compartments is consistent with an EMT program engaged wherever nephron-forming cells undergo state transitions; its persistence in dysplastic kidneys, in which orderly induction fails, suggests that this machinery is deployed but is spatially miscoordinated rather than absent. Because SNAI1 is normally silenced as the mesenchyme epithelializes and is pathogenic when reactivated [11], and because even a partial, non-cell-autonomous EMT program is sufficient to reshape renal tissue in disease models [30,31], an increase in the spatial coupling of SNAIL and TGF-β1 without a change in their abundance is most economically read as a shift in where the program is engaged rather than in how much of it is expressed. The medulla-restricted rise in CD31–vimentin coupling, by contrast, marks the compartment in which endothelial and interstitial populations interconvert, and is compatible with engagement of an endothelial-to-mesenchymal axis, although CD31–vimentin co-occurrence is not specific to EndMT and cannot, alone, distinguish transition from the normal co-residence of endothelial and vimentin-positive mesenchymal cells; lineage-based approaches would be required to establish EndMT [15,32].

That the two programs dissociate argues against a technical explanation. A nonspecific artifact of staining, imaging, or thresholding would inflate colocalization indiscriminately, affecting both pairs, in all compartments, in all groups. Instead, one pair shifts throughout the CAKUT spectrum while the other shifts only in the medulla, and neither varies across developmental phases in control kidneys. This specificity is difficult to reconcile with a generic measurement bias and is more readily explained by a genuine difference in how these gene products are distributed within malformed tissue.

Taken together, these observations point to a model in which the disruption of nephrogenesis in CAKUT is reflected in tissue-level reorganization of otherwise intact transcriptional programs, rather than in their upregulation or downregulation. Unchanged transcript and protein levels, alongside altered spatial coupling, imply regulation downstream of transcription—at the level of protein localization, cellular positioning, and tissue organization. That the SNAIL–TGF-β1 shift persists even in dysplastic kidneys, in which architecture is grossly disrupted, indicates that this coupling accompanies the malformed state itself rather than being a passive consequence of preserved tissue geometry.

This has a practical implication for the genetic analysis of malformation. Differential expression analysis and intensity-based quantification—the standard readouts—may be systematically blind to this class of changes. Had we relied on either alone, we would have concluded that these programs are unaltered in CAKUT. Colocalization analysis, which is straightforward to add to any double-labeling experiment, revealed a difference that neither could detect, and provides a readout linking malformation to the in situ organization of gene products.

### Limitations

Several limitations must be acknowledged. First, the cohort is small, as is inherent to studies of human fetal malformed kidney tissue; the compartment-specific analysis rests on nine architecturally preserved CAKUT kidneys, and individual phenotype subgroups (*n =* 2–4) are too small to support phenotype-level inference, which we present as exploratory only. Because the late-gestation group was small (Ph4, *n* = 2), trends in late nephrogenesis cannot be excluded; the analysis indicates the absence of a detectable difference rather than proving equivalence. Developmental age differed between groups (control median 21.5 vs. CAKUT 30.5 weeks; Mann–Whitney U, *p* = 0.004); because abundance and colocalization did not vary with developmental phase (Table S5), this is unlikely to confound the comparisons. Second, the CAKUT phenotypes pooled for the main comparison are genetically and biologically heterogeneous, and genotype was not available for the specimens analyzed; we therefore cannot relate the observed reorganization to specific pathogenic variants, which remains an important objective for future work. Third, although four of six primary comparisons survived correction for multiple testing, effect sizes were moderate (median shifts in Pearson’s coefficient of approximately 0.06–0.09 within an already high range). Fourth, the transcriptomic datasets analyzed are derived from ureteric tissue (human) and adult kidney (mouse) rather than from the same fetal renal specimens used for immunofluorescence, so transcript and protein data are concordant but not directly paired. Fifth, quantification was performed at the level of the specimen rather than the image; this avoids pseudoreplication but limits power. Finally, colocalization at conventional fluorescence resolution indicates spatial proximity, not molecular interaction; functional studies would be required to establish whether the increased coupling reflects genuine signaling cross-talk.

## 5. Conclusions

In human fetal kidneys with CAKUT, the EMT and vascular–mesenchymal gene programs are not quantitatively activated: SNAI1, TGFB1–3, PECAM1 and VIM are expressed at control levels at both transcript and protein levels, with no significant change across the phases sampled. What is altered is the spatial deployment of their products. Tighter SNAIL–TGF-β1 coupling is a consistent feature across the CAKUT spectrum, being present in the cortex, the medulla, and in dysplastic kidneys alike, whereas increased CD31–vimentin coupling is most evident in the medulla. Disruption of nephrogenesis in the malformed state may therefore be reflected in tissue-level reorganization of intact transcriptional programs, a difference invisible to differential expression analysis and to abundance-based quantification, but readily revealed by colocalization analysis of gene products in situ.

## Figures and Tables

**Table 1:** Primary and secondary antibodies used for immunofluorescence staining.

Antibody		Catalog Number	Host	Dilution	Source
Primary	CD31 (PECAM-1) (89C2)	#3528S	Mouse	1:400	Cell Signaling Technology (CST), (Danvers, MA, USA)
	Vimentin (D21H3) XP^®^ mAb	#5741S	Rabbit	1:300	Cell Signaling Technology (CST), (Danvers, MA, USA)
	Snail (L70G2) Mouse Monoclonal Antibody	CST 3895	Mouse	1:200	Cell Signaling Technology (CST), (Danvers, MA, USA)
	TGF Beta 1 antibody	21898-1-AP	Rabbit	1:300	Proteintech Group, Inc. (Rosemont, IL, USA)
Blocking antigen (preadsorption)	Recombinant human CD31 protein (aa 28–601)	ab155607	Recombinant protein (HEK293)	N/A	Abcam (Cambridge, UK)
	Vimentin (D21H3) blocking peptide	ab46156	Synthetic peptide, >90% HPLC	N/A	Abcam (Cambridge, UK)
	Snail (L70G2)	CDBP2743	Synthetic peptide, lyophilized	N/A	Creative Diagnostics (Shirley, NY, USA)
	TGF Beta 1	MBS823587	Synthetic peptide	N/A	MyBioSource (San Diego, CA, USA)
Secondary	Alexa Fluor^®^ 488 AffiniPure™ Donkey Anti-Mouse IgG (H + L)	715-545-150	Donkey	1:400	Jackson Immuno Research Laboratories, Inc. (West Grove, PA, USA)
	Rhodamine Red™-X (RRX) AffiniPure™ Donkey Anti-Rabbit IgG (H + L)	711-295-152	Donkey	1:400	Jackson Immuno Research Laboratories, Inc. (West Grove, PA, USA)

## Data Availability

The transcriptomic datasets analyzed in this study are publicly available in the NCBI Gene Expression Omnibus (https://www.ncbi.nlm.nih.gov/geo/; accessed on 12 June 2026) under accession numbers GSE83946 [17] and GSE42303 [18]. The immunofluorescence data generated in this study are available from the corresponding author upon reasonable request.

## Supplementary Materials

The following supporting information can be downloaded at https://www.mdpi.com/article/10.3390/genes17091028/s1. Table S1. The samples of human embryonic and fetal kidneys (*n* = 38) analyzed in the study; Table S2. Complete statistical results (nominal *p* and BH-adjusted q values); Table S3. Per-phenotype exploratory comparisons (whole-specimen values). Interpret with caution: *n =* 2–4 per phenotype.; Table S4. Secondary colocalization metrics and Costes randomization; Table S5. Whole-specimen abundance analysis and developmental regression (control kidneys); Table S6. Transcriptomic reanalysis of publicly available datasets (R/limma, Benjamini–Hochberg correction); Table S7. Specimen-level marker abundance—Cortex (% signal area); Table S8. Specimen-level marker abundance—Medulla (% signal area); Table S9. Specimen-level colocalization (Pearson’s coefficient); Code S1. Analysis code (R). Supplementary_analysis_code.R reproduces the transcriptomic reanalysis (GSE83946, 7 controls vs. 19 CAKUT; GSE42303, wild-type only, 3 sham vs. 3 UUO) and Figure 1, and the immunofluorescence statistics (Hodges–Lehmann effect sizes with 95% CIs, developmental-age comparison, and the group × compartment linear mixed-effects model). Differential expression used limma with quantile normalization and Benjamini–Hochberg FDR control.

## Abbreviations

The following abbreviations are used in this manuscript.

AICc	corrected Akaike information criterion
ANOVA	analysis of variance
CAKUT	congenital anomalies of the kidney and urinary tract
CD31	cluster of differentiation 31 (protein product of PECAM1)
CKD	chronic kidney disease
DAPI	4′,6-diamidino-2-phenylindole
EMT	epithelial–mesenchymal transition
EndMT	endothelial-to-mesenchymal transition
FDR	false discovery rate
FFPE	formalin-fixed, paraffin-embedded
GEO	Gene Expression Omnibus
H&E	hematoxylin and eosin
ICC	intraclass correlation coefficient
IF	immunofluorescence
IQR	interquartile range
log_2_FC	log2 fold change
MET	mesenchymal-to-epithelial transition
PBS	phosphate-buffered saline
PECAM1	platelet and endothelial cell adhesion molecule 1
SNAI1	snail family transcriptional repressor 1 (protein: SNAIL)
TGF-β (TGFB)	transforming growth factor beta
UUO	unilateral ureteral obstruction
VIM	vimentin
