# Supplementary material for: Transcript- and Protein-Level Preservation and Spatial Reorganization of EMT and Vascular–Mesenchymal Gene Programs (SNAI1, TGFB1, PECAM1, VIM) in Human Fetal Kidneys with Congenital Anomalies of the Kidney and Urinary Tract (CAKUT)
Source: Genes (Basel). 2026 Aug 28;17(9):1028. doi: 10.3390/genes17091028 (PMC13606875; doi:10.3390/genes17091028)
Supplement: Supplementary file 1 [file genes-17-01028-s001.zip › genes-4507649-supplementary.pdf]

Table S1. The samples of human embryonic and fetal kidneys (n=38) analyzed in the study

Groups	Developmental phase	Renal and associated pathology	Gestational phases	Number of kidney samples
Normal kidneys (CTRL)*	Ph1	N/A	14	1
	Ph2	N/A	15	1
		N/A	15-16	1
		N/A	16	2
		N/A	18	2
		N/A	21	2
		N/A	21-22	1
	Ph3	N/A	23	1
		N/A	24	1
		N/A	28	3
		N/A	32	1
		N/A	35	1
	Ph4	N/A	37	1
		N/A	38	1
Horseshoe kidney (HK)	*Ren concreatus arcuatus, cystae multiplices corticales*		22	1
	Ren concreatus arcuatus, tetras Fallot		26	1
	Syndroma Edwards, Ren arcuatus		30-31	1
	Syndroma Edwards, Ren arcuatus		34	1
Dysplastic kidneys (DYS)	*Megaureter lateris dextri, Dysplasia renis*		21	1
	Renis dysplastica cysticus lateralis sinistri, agenesia renis dextri		22	1
	Dysplasia multicystica renis dextri<br>*Cystes parvae focales*		27	1
	Dysplasia renis		28	1
	Renes dysplastici cystici,<br>*Syndroma Potter*		35	2
	Dysplasia renis multicystica bilateralis		36	1
	Agenesis renis dextri et dysplasia renis sinistri cum ureter duplex		37	1
	Dysplasia hypoplastica, renis bilateralis, syndroma Down, syndroma Potter		38	1
	Syndroma Potter, Dysplasia renis		39	1
Hypoplastic kidneys (HYP)	*Hypoplasia renis lateris dextri*		30	1
	Hypoplasia renis lateris sinistri		37	1

	Hypoplasia renis	38	1
Duplex kidneys (DK)	*Ureter duplex lateris dextri*	24	1
	Ureter duplex lateris sinistri	30	1

*One control without recorded developmental age was included in the abundance analyses (total control n = 20).

Table S2. Complete statistical results (nominal p and BH-adjusted q values)

A. Colocalization (Pearson's coefficient) — primary comparisons							
Marker pair	Comparison	Control median (n)	CAKUT median (n)	Test	p	q (BH)	Significant
SNAIL–TGF-β	Control vs CAKUT — cortex	0.740 (n=19)	0.833 (n=9)	Mann–Whitney U	0,0159	0,0319	Yes
SNAIL–TGF-β	Control vs CAKUT — medulla	0.779 (n=19)	0.836 (n=9)	Mann–Whitney U	0,0236	0,0355	Yes
SNAIL–TGF-β	Control vs dysplasia (whole tissue)	0.757 (n=19)	0.850 (n=10)	Mann–Whitney U	0,0083	0,0316	Yes
CD31–vimentin	Control vs CAKUT — cortex	0.793 (n=19)	0.863 (n=9)	Mann–Whitney U	0,0616	0,0739	No
CD31–vimentin	Control vs CAKUT — medulla	0.770 (n=19)	0.848 (n=9)	Mann–Whitney U	0,0105	0,0316	Yes
CD31–vimentin	Control vs dysplasia (whole tissue)	0.765 (n=19)	0.825 (n=10)	Mann–Whitney U	0,1134	0,1134	No

B. Colocalization — pooled analysis (cortex + dysplastic whole tissue)					
Marker pair	Comparison	Control median (n)	CAKUT median (n)	Test	p
SNAIL–TGF-β	Control vs CAKUT (pooled)	0.740 (n=19)	0.839 (n=19)	Mann–Whitney U	0,0055
CD31–vimentin	Control vs CAKUT (pooled)	0.793 (n=19)	0.841 (n=19)	Mann–Whitney U	0,0659

C. Marker abundance (% signal area) — Control vs CAKUT								
Marker	Compartment	Control median (n)	CAKUT median (n)	Shapiro-Wilk (ctrl / CAKUT)	Test	p	Median difference (HL, CAKUT−Control)	95% CI
SNAIL	Cortex	0.142 (n=20)	0.111 (n=9)	0.055 / 0.905	Welch's t-test	0,2590	−0.017	−0.058 to +0.027
SNAIL	Medulla	0.097 (n=20)	0.098 (n=9)	0.000 / 0.220	Mann–Whitney U	0,8136	−0.003	−0.056 to +0.041

TGF-β	Cortex	0.947 (n=20)	1.223 (n=9)	0.003 / 0.428	Mann–Whitney U	0,5245	+0.163	−0.269 to +0.610
TGF-β	Medulla	1.380 (n=20)	1.435 (n=9)	0.002 / 0.161	Mann–Whitney U	0,4367	+0.110	−0.340 to +0.652
CD31	Cortex	0.262 (n=18)	0.199 (n=9)	0.000 / 0.000	Mann–Whitney U	0,4558	−0.048	−0.171 to +0.152
CD31	Medulla	0.376 (n=18)	0.222 (n=9)	0.012 / 0.002	Mann–Whitney U	0,2076	−0.098	−0.317 to +0.080
VIM	Cortex	0.601 (n=19)	0.681 (n=9)	0.000 / 0.329	Mann–Whitney U	0,2579	+0.084	−0.062 to +0.263
VIM	Medulla	0.593 (n=19)	0.717 (n=9)	0.000 / 0.824	Mann–Whitney U	0,3252	+0.115	−0.120 to +0.377

Effect sizes are Hodges–Lehmann median differences (CAKUT − Control) with 95% confidence intervals; all intervals include zero, consistent with the absence of a detectable difference.

Table S3. Per-phenotype exploratory comparisons (whole-specimen values). Interpret with caution: n = 2–4 per phenotype.

A. Marker abundance (% signal area)						
Marker	Control	Ureteric anomaly	Hypoplasia	Horseshoe kidney	Dysplasia	Kruskal–Wallis p
SNAIL	0.133 (n=20)	0.098 (n=2)	0.137 (n=3)	0.097 (n=4)	0.154 (n=10)	0,1771
TGF-β	0.947 (n=20)	1.216 (n=2)	1.679 (n=3)	0.941 (n=4)	1.183 (n=10)	0,1739
CD31	0.285 (n=18)	0.310 (n=2)	0.215 (n=3)	0.229 (n=4)	0.218 (n=10)	0,7132
VIM	0.601 (n=19)	0.595 (n=2)	0.681 (n=3)	0.773 (n=4)	0.749 (n=10)	0,6683
B. Colocalization (Pearson's coefficient)						
Marker pair	Control	Ureteric anomaly	Hypoplasia	Horseshoe	Dysplasia	Kruskal–Wallis p
SNAIL–TGF-β	0.740 (n=19)	0.819 (n=2)	0.854 (n=3)	0.804 (n=4)	0.850 (n=10)	0,0637
CD31–vimentin	0.793 (n=19)	0.842 (n=2)	0.875 (n=3)	0.804 (n=4)	0.825 (n=10)	0,1709

Cortical values for architecturally preserved kidneys; whole-tissue values for dysplasia. Phenotype subgroups (n = 2–4) are underpowered; results are exploratory.

Table S4. Secondary colocalization metrics and Costes randomization

A. Group comparisons for secondary metrics (Mann–Whitney U)					
Marker pair	Compartment	Metric	Control median	CAKUT median	p
SNAIL–TGF-β	Cortex	Spearman's ϱ	0,751	0,846	0,0121
SNAIL–TGF-β	Cortex	Manders' M1	0,878	0,944	0,0068
SNAIL–TGF-β	Cortex	Manders' M2	0,911	0,953	0,0491
SNAIL–TGF-β	Cortex	Overlap coefficient	0,904	0,939	0,0047
SNAIL–TGF-β	Medulla	Spearman's ϱ	0,726	0,808	0,0236
SNAIL–TGF-β	Medulla	Manders' M1	0,854	0,929	0,0121
SNAIL–TGF-β	Medulla	Manders' M2	0,897	0,933	0,0437
SNAIL–TGF-β	Medulla	Overlap coefficient	0,903	0,933	0,0304
CD31–vimentin	Cortex	Spearman's ϱ	0,706	0,795	0,0286

CD31–vimentin	Cortex	Manders' M1	0,871	0,926	0,0182
CD31–vimentin	Cortex	Manders' M2	0,883	0,932	0,0304
CD31–vimentin	Cortex	Overlap coefficient	0,913	0,944	0,0550
CD31–vimentin	Medulla	Spearman's ϱ	0,683	0,815	0,0121
CD31–vimentin	Medulla	Manders' M1	0,848	0,926	0,0079
CD31–vimentin	Medulla	Manders' M2	0,850	0,938	0,0063
CD31–vimentin	Medulla	Overlap coefficient	0,905	0,937	0,0105
B. Field exclusion due to failure of Costes automatic thresholding (threshold > 30)					
Marker pair	Total fields	Fields retained	Fields excluded	% excluded	
SNAIL–TGF-β	276	266	10	3,6	
CD31–vimentin	281	263	18	6,4	

Costes' randomization (200 iterations) confirmed non-random colocalization in every specimen analysed. Manders' coefficients are threshold-dependent and are therefore reported as secondary metrics only.

Table S5. Whole-specimen abundance analysis and developmental regression (control kidneys)

A. Whole-specimen abundance across groups (field-weighted mean of all imaged fields)						
Marker	Control	Ureteric anomaly	Hypoplasia	Horseshoe	Dysplasia	Kruskal–Wallis p
SNAIL	0.133 (n=20)	0.098 (n=2)	0.137 (n=3)	0.097 (n=4)	0.154 (n=10)	0,1771
TGF-β	0.947 (n=20)	1.216 (n=2)	1.679 (n=3)	0.941 (n=4)	1.183 (n=10)	0,1739
CD31	0.285 (n=18)	0.310 (n=2)	0.215 (n=3)	0.229 (n=4)	0.218 (n=10)	0,7132
VIM	0.601 (n=19)	0.595 (n=2)	0.681 (n=3)	0.773 (n=4)	0.749 (n=10)	0,6683
B. Abundance across developmental phases (control kidneys)						
Marker	Compartment	Ph2 median (n)	Ph3 median (n)	Ph4 median (n)	Kruskal–Wallis p	
SNAIL	Cortex	0.123 (n=9)	0.139 (n=7)	0.150 (n=2)	0,6756	
SNAIL	Medulla	0.102 (n=9)	0.090 (n=7)	0.108 (n=2)	0,3429	
TGF-β	Cortex	0.838 (n=9)	0.890 (n=7)	1.744 (n=2)	0,2335	
TGF-β	Medulla	0.806 (n=9)	1.431 (n=7)	1.266 (n=2)	0,3003	
CD31	Cortex	0.212 (n=9)	0.262 (n=6)	0.419 (n=2)	0,3207	
CD31	Medulla	0.395 (n=9)	0.265 (n=6)	0.507 (n=2)	0,1314	
VIM	Cortex	0.529 (n=9)	0.626 (n=6)	0.651 (n=2)	0,5596	
VIM	Medulla	0.511 (n=9)	0.686 (n=6)	0.588 (n=2)	0,4752	
*C. Regression of abundance on developmental phase (control kidneys, cortex)						
Marker	n	Linear slope β ± SE	R^2 (linear)	p (slope = 0)	R^2 (quadratic)	Preferred model (AICc)
SNAIL	18	+0.0070 ± 0.0269	0.004	0.799	0.005	Linear
TGF-β	18	+0.121 ± 0.260	0.013	0.647	0.111	Linear
CD31	17	−0.046 ± 0.145	0.007	0.752	0.019	Linear
VIM	17	−0.033 ± 0.169	0.003	0.847	0.003	Linear

*No marker showed a significant linear or quadratic trend with developmental phase.

Table S6. Transcriptomic reanalysis of publicly available datasets (R/limma, Benjamini–Hochberg correction)

*A. GSE83946 — human CAKUT ureteric tissue (7 controls, 19 CAKUT)						
IF marker	Gene	log2FC	p	FDR (BH)	Detection (ctrl / case)	Regulation
SNAIL	*SNAI1*	−2.16	0.030	0.340	14% / 26%	Uninformative (below detection limit)
TGF-β1	*TGFB1*	+0.96	0.456	0.819	43% / 58%	n.s.
TGF-β2	*TGFB2*	−0.36	0.485	0.832	100% / 100%	n.s.
TGF-β3	*TGFB3*	−0.32	0.133	0.572	100% / 100%	n.s.
CD31	*PECAM1*	+0.52	0.012	0.245	100% / 100%	↑ nominal only (not FDR-significant)
Vimentin	*VIM*	+0.36	0.028	0.330	100% / 100%	↑ nominal only (not FDR-significant)
†B. GSE42303 — murine unilateral ureteral obstruction (3 sham, 3 UUO)						
IF marker	Gene	log2FC	p	WT-only FDR	Detection (sham / UUO)	Regulation
SNAIL	*Snai1*	+0.52	0.158	0.190	100% / 100%	n.s.
TGF-β1	*Tgfb1*	+1.34	0.0022	0.0066	100% / 100%	↑
TGF-β2	*Tgfb2*	+1.30	0.0047	0.0071	100% / 100%	↑
TGF-β3	*Tgfb3*	+2.63	0.0065	0.0087	100% / 100%	↑
CD31	*Pecam1*	+0.78	0.043	0.043	100% / 100%	↑
Vimentin	*Vim*	+2.82	0.0005	0.0031	100% / 100%	↑

*No gene survived FDR correction. SNAI1 was detected in a minority of samples and close to the detection limit; its apparent decrease is not interpretable.

†These programmes are strongly inducible in renal tissue following acquired obstructive injury, yet are not transcriptionally activated in human congenital malformation (panel A).

Table S7. Specimen-level marker abundance — Cortex (% signal area)

#	SNAIL Ctrl	SNAIL CAKUT	TGF-β1 Ctrl	TGF-β1 CAKUT	CD31 Ctrl	CD31 CAKUT	VIM Ctrl	VIM CAKUT
1	0.025	0.106	0.407	0.784	0.332	0.073	1.091	0.663
2	0.123	0.083	3.290	1.647	0.055	0.705	2.345	0.954
3	0.076	0.052	0.704	1.809	0.221	0.098	0.410	0.576
4	0.145	0.154	0.855	1.314	0.187	0.408	0.630	0.883
5	0.080	0.087	1.031	1.679	0.279	0.199	0.545	0.484
6	0.116	0.121	0.455	0.592	0.184	1.860	0.485	0.706
7	0.192	0.205	0.756	1.179	0.154	0.247	0.743	0.681
8	0.134	0.149	0.838	0.704	0.721	0.163	0.562	0.965
9	0.139	0.111	1.379	1.223	0.269	0.181	0.529	0.585
10	0.157		2.289		0.212		0.492	
11	0.155		1.222		0.493		1.226	
12	0.167		0.800		0.245		0.889	
13	0.081		1.516		0.337		0.409	
14	0.145		0.621		0.345		0.413	
15	0.128		1.745		0.256		0.601	
16	0.253		0.558		1.230		0.366	
17	0.194		0.890		1.537		0.626	
18	0.064		1.743		0.187		0.702	
19	0.090		1.053				0.626	

20	0.354		1.004					
n	20	9	20	9	18	9	19	9
Median	0.136	0.111	0.947	1.223	0.263	0.199	0.601	0.681

Values are per-specimen area percentages (mean of imaged fields). n and median per group are shown at the foot of each column.

Table S8. Specimen-level marker abundance — Medulla (% signal area)

#	SNAIL Ctrl	SNAIL CAKUT	TGF-β1 Ctrl	TGF-β1 CAKUT	CD31 Ctrl	CD31 CAKUT	VIM Ctrl	VIM CAKUT
1	0.057	0.098	0.529	1.436	0.118	0.066	0.733	1.188
2	0.134	0.111	3.918	1.457	0.094	0.157	1.242	0.665
3	0.093	0.027	0.715	2.218	0.755	0.043	0.654	0.582
4	0.074	0.250	0.899	0.911	0.384	0.460	0.551	1.012
5	0.053	0.067	1.776	2.681	0.999	0.255	1.398	0.354
6	0.185	0.098	0.521	0.800	0.125	1.332	0.416	0.833
7	0.154	0.042	1.431	1.229	0.237	0.222	0.482	0.535
8	0.100	0.105	0.806	1.143	0.371	0.067	0.744	1.111
9	0.090	0.153	1.871	1.437	0.233	0.349	0.387	0.717
10	0.557		1.349		0.085		0.510	
11	0.061		2.058		0.428		1.806	
12	0.103		0.998		0.297		0.693	
13	0.042		1.430		0.181		0.535	
14	0.154		0.724		0.586		0.511	
15	0.149		1.031		0.380		0.550	
16	0.071		0.701		1.072		0.340	
17	0.102		1.411		0.395		0.680	
18	0.094		1.501		0.410		0.627	
19	0.031		2.036				0.593	
20	0.260		1.566					
n	20	9	20	9	18	9	19	9
Median	0.097	0.098	1.380	1.436	0.376	0.222	0.593	0.717

Values are per-specimen area percentages. n and median per group are shown at the foot of each column.

Table S9. Specimen-level colocalization (Pearson's coefficient)

#	SNAIL–TGF-β Cortex Ctrl	SNAIL–TGF-β Cortex CAKUT	SNAIL–TGF-β Medulla Ctrl	SNAIL–TGF-β Medulla CAKUT	CD31–vimentin Cortex Ctrl	CD31–vimentin Cortex CAKUT	CD31–vimentin Medulla Ctrl	CD31–vimentin Medulla CAKUT
1	0.505	0.765	0.518	0.666	0.470	0.706	0.376	0.781
2	0.590	0.775	0.534	0.741	0.475	0.795	0.593	0.807
3	0.648	0.816	0.671	0.782	0.566	0.813	0.676	0.814
4	0.654	0.821	0.673	0.811	0.674	0.821	0.713	0.845
5	0.687	0.833	0.677	0.836	0.685	0.863	0.717	0.848
6	0.689	0.839	0.695	0.847	0.713	0.872	0.724	0.907
7	0.701	0.854	0.706	0.867	0.735	0.875	0.736	0.911
8	0.728	0.896	0.715	0.871	0.738	0.891	0.747	0.929
9	0.737	0.918	0.746	0.961	0.768	0.899	0.767	0.946
10	0.740		0.779		0.793		0.770	
11	0.773		0.779		0.800		0.782	

12	0.797		0.779		0.818		0.795	
13	0.800		0.786		0.819		0.807	
14	0.805		0.791		0.828		0.826	
15	0.825		0.791		0.833		0.836	
16	0.834		0.807		0.845		0.875	
17	0.850		0.809		0.875		0.892	
18	0.873		0.823		0.887		0.907	
19	0.886		0.855		0.900		0.937	
n	19	9	19	9	19	9	19	9
Median	0.740	0.833	0.779	0.836	0.793	0.863	0.770	0.848

Per-specimen Pearson's colocalization coefficients for the SNAIL–TGF-β and CD31–vimentin pairs, by compartment

Code S1. Analysis code (R). Supplementary_analysis_code.R reproduces the transcriptomic reanalysis (GSE83946, 7 controls vs 19 CAKUT; GSE42303, wild-type only, 3 sham vs 3 UUO) and Figure 1, and the immunofluorescence statistics (Hodges–Lehmann effect sizes with 95% CIs, developmental-age comparison, and the group × compartment linear mixed-effects model). Differential expression used limma with quantile normalization and Benjamini–Hochberg FDR control.

```
################################################################################
#   Supplementary analysis code   —   genes-4507649
#   "Transcript- and Protein-Level Preservation and Spatial Reorganization of
#     EMT and Vascular-Mesenchymal Gene Programs in Human Fetal Kidneys with CAKUT"
#
#   Contents
#      1. Transcriptomic reanalysis (GEO) and Figure 1
#           - GSE83946 : human CAKUT ureteric tissue   (7 control vs 19 CAKUT)
#           - GSE42303 : murine UUO kidney, wild-type only (3 sham vs 3 UUO)
#      2. Immunofluorescence statistics (from specimen-level CSV files)
#           - Effect sizes + 95% CI for marker abundance (Hodges-Lehmann)
#           - Developmental age comparison (control vs CAKUT)
#           - Group x compartment interaction (linear mixed-effects model)
#
#   Input data files (provided as Supplementary):
#      abundance.csv : marker, compartment, group, value        (area %, per field-averaged specimen)
#      coloc.csv        : specimen, group, compartment, pair, pearson
#      cohort.csv       : specimen, group, week, sex, diagnosis   (optional; for age test)
#
#   Run:   source("Supplementary_analysis_code.R")
################################################################################

## ---------------------------------------------------------------- 0. SETUP
OUTDIR <- "analysis_out"; dir.create(OUTDIR, showWarnings = FALSE)
```

```r
pkgs <- c("GEOquery","limma","ggplot2","dplyr","stringr","patchwork","lmerTest")
for (p in pkgs) {
  if (!requireNamespace(p, quietly = TRUE)) {
    if (p %in% c("GEOquery","limma")) {
      if (!requireNamespace("BiocManager", quietly = TRUE)) install.packages("BiocManager")
      BiocManager::install(p, ask = FALSE, update = FALSE)
    } else install.packages(p, repos = "https://cloud.r-project.org")
  }
}
suppressPackageStartupMessages({
  library(GEOquery); library(limma); library(ggplot2); library(dplyr)
  library(stringr);   library(patchwork); library(lmerTest)
})

## ==========================================================================
## 1. TRANSCRIPTOMIC REANALYSIS
## ==========================================================================
markers <- data.frame(
  human = c("PECAM1","SNAI1","TGFB1","TGFB2","TGFB3","VIM"),
  mouse = c("Pecam1","Snai1","Tgfb1","Tgfb2","Tgfb3","Vim"),
  stringsAsFactors = FALSE)

# --- helpers ----------------------------------------------------------------
symbol_col <- function(eset) {
  fd <- fData(eset)
  col <- names(fd)[str_detect(tolower(names(fd)), "gene.?symbol|^symbol$|gene_symbol")][1]
  if (is.na(col)) stop("No gene-symbol column in fData().")
  col
}
# per-gene long expression (highest-mean probe where several map to one gene)
expr_long <- function(eset, symbols) {
  fd   <- fData(eset); syms <- toupper(trimws(as.character(fd[[symbol_col(eset)]])))
  mat <- exprs(eset)
  do.call(rbind, lapply(symbols, function(g) {
    idx <- which(syms == toupper(g)); if (!length(idx)) return(NULL)
    if (length(idx) > 1) idx <- idx[which.max(rowMeans(mat[idx,,drop=FALSE], na.rm = TRUE))]
    data.frame(gene = g, expr = as.numeric(mat[idx,]), sample = colnames(mat))
  }))
}
# full limma table (log2FC, nominal p, BH-FDR) for the requested genes
limma_table <- function(eset, grp, symbols) {
  grp <- factor(grp, levels = c("Control","Case"))
  fit <- eBayes(lmFit(exprs(eset), model.matrix(~ grp)))
  tt   <- topTable(fit, coef = 2, number = Inf, sort.by = "none")
```

```
  tt$SYM <- toupper(trimws(as.character(fData(eset)[[symbol_col(eset)]])))
  do.call(rbind, lapply(symbols, function(g) {
    s <- tt[tt$SYM == toupper(g), , drop = FALSE]
    if (!nrow(s)) return(data.frame(gene = g, log2FC = NA, p = NA, FDR = NA))
    s <- s[which.min(s$adj.P.Val), ]
    data.frame(gene = g, log2FC = round(s$logFC,3), p = signif(s$P.Value,3), FDR = signif(s$adj.P.Val,3))
  }))
}
sig_star <- function(fdr) ifelse(is.na(fdr),"ns",ifelse(fdr<0.001,"***",ifelse(fdr<0.01,"**",ifelse(fdr<0.05,"*","ns"))))

pal <- c(Control = "#CFCAC2", Case = "#9EC1B5")
make_panel <- function(long, fdr, order, title, case_label) {
  long$gene   <- factor(long$gene, levels = order)
  long$group <- factor(long$group, levels = c("Control","Case"))
  rng <- long %>% group_by(gene) %>%
    summarise(hi = max(expr,na.rm=TRUE), lo = min(expr,na.rm=TRUE), .groups="drop")
  rng$y <- rng$hi + 0.12*(rng$hi-rng$lo); rng$lab <- sig_star(fdr[as.character(rng$gene)])
  ggplot(long, aes(group, expr, fill = group)) +
    geom_boxplot(outlier.shape=NA, alpha=.85, colour="grey45", linewidth=.4, width=.6) +
    geom_jitter(width=.15, size=.7, alpha=.45, colour="grey35") +
    geom_text(data=rng, aes(1.5, y, label=lab), inherit.aes=FALSE, size=4) +
    facet_wrap(~gene, scales="free_y", nrow=2) +
    scale_x_discrete(labels=c(Control="Control", Case=case_label)) +
    scale_fill_manual(values=c(Control=unname(pal["Control"]), Case=unname(pal["Case"]))) +
    labs(title=title, x=NULL, y="log2 expression") +
    theme_bw(base_size=11) +
    theme(legend.position="none", strip.text=element_text(face="italic"),
          panel.grid.minor=element_blank())
}

## --- GSE83946 : human (7 control vs 19 CAKUT) ------------------------------
e1 <- getGEO("GSE83946", destdir=OUTDIR, GSEMatrix=TRUE, AnnotGPL=TRUE)[[1]]
m1 <- exprs(e1); if (max(m1,na.rm=TRUE) > 100) m1 <- log2(m1+1)
exprs(e1) <- normalizeBetweenArrays(m1, method="quantile")
ph1 <- pData(e1)
info1 <- ph1[, grep("characteristics|source_name|^title$", tolower(names(ph1))), drop=FALSE]
txt1 <- apply(info1, 1, function(r) paste(tolower(r), collapse=" | "))
grp1 <- ifelse(str_detect(txt1, "control|non[- ]?cakut|healthy|normal ureter"), "Control", "Case")
cat("GSE83946 groups: "); print(table(grp1))          # expected Control 7 / Case 19
tabH <- limma_table(e1, grp1, markers$human)
cat("\n== GSE83946 (human, 7 vs 19) ==\n"); print(tabH)
L1 <- expr_long(e1, markers$human); L1$group <- grp1[match(L1$sample, rownames(ph1))]
pA <- make_panel(L1, setNames(tabH$FDR, tabH$gene), markers$human, "GSE83946 \u2014 human CAKUT (ure-
ter)", "CAKUT")
```

```
## --- GSE42303 : mouse, WILD-TYPE ONLY (3 sham vs 3 UUO) --------------------
e2 <- getGEO("GSE42303", destdir=OUTDIR, GSEMatrix=TRUE, AnnotGPL=TRUE)[[1]]
m2 <- exprs(e2); if (max(m2,na.rm=TRUE) > 100) m2 <- log2(m2+1)
exprs(e2) <- normalizeBetweenArrays(m2, method="quantile")
ph2 <- pData(e2)
info2 <- ph2[, grep("characteristics|source_name|^title$", tolower(names(ph2))), drop=FALSE]
txt2 <- apply(info2, 1, function(r) paste(tolower(r), collapse=" | "))
wt     <- str_detect(txt2, "wild|wt|\\+/\\+|wild.?type")                    # keep wild-type only
e2wt <- e2[, wt]
grp2 <- ifelse(str_detect(txt2[wt], "sham"), "Control", "Case") # sham=Control, UUO=Case
cat("\nGSE42303 wild-type groups: "); print(table(grp2))                # expected 3 / 3
tabM <- limma_table(e2wt, grp2, markers$mouse)
cat("\n== GSE42303 (mouse, wild-type only, 3 vs 3) ==\n"); print(tabM)
L2 <- expr_long(e2wt, markers$mouse); L2$group <- grp2[match(L2$sample, colnames(e2wt))]
pB <- make_panel(L2, setNames(tabM$FDR, tabM$gene), markers$mouse, "GSE42303 \u2014 mouse UUO (kidney, wild-type)", "UUO")

fig1 <- (pA / pB) + plot_annotation(tag_levels = "a")
ggsave(file.path(OUTDIR,"Figure_1.tif"), fig1, width=6, height=8.4, dpi=300, compression="lzw")
cat("\n[OK] Figure 1 saved.\n")

## ============================================================================
## 2. IMMUNOFLUORESCENCE STATISTICS (specimen-level CSV files)
## ============================================================================
## --- 2a. Abundance: effect size (Hodges-Lehmann) + 95% CI ------------------
if (file.exists("abundance.csv")) {
  ab <- read.csv("abundance.csv")
  hl <- function(d) {
    x <- d$value[d$group=="CAKUT"]; y <- d$value[d$group=="Control"]
    w <- suppressWarnings(wilcox.test(x, y, conf.int=TRUE, exact=FALSE))
    data.frame(HL_diff=round(unname(w$estimate),4),
               CI_low=round(w$conf.int[1],4), CI_high=round(w$conf.int[2],4),
               p=round(w$p.value,4))
  }
  eff <- ab %>% group_by(marker, compartment) %>% group_modify(~hl(.x)) %>% ungroup()
  cat("\n== Abundance effect sizes (Hodges-Lehmann, 95% CI) ==\n"); print(as.data.frame(eff))
  write.csv(eff, file.path(OUTDIR,"abundance_effect_sizes.csv"), row.names=FALSE)
}

## --- 2b. Developmental age: control vs CAKUT ------------------------------
if (file.exists("cohort.csv")) {
  co <- read.csv("cohort.csv")
  a <- suppressWarnings(wilcox.test(week ~ group, data=co, exact=FALSE))
```

```
  cat("\n== Developmental age (control vs CAKUT): Mann-Whitney p =", round(a$p.value,3), "==\n")
}

## --- 2c. Group x compartment interaction (mixed-effects) ------------------
if (file.exists("coloc.csv")) {
  cc <- read.csv("coloc.csv")
  for (pr in unique(cc$pair)) {
    d <- droplevels(subset(cc, pair==pr))
    d$group <- factor(d$group, levels=c("Control","CAKUT"))
    d$compartment <- factor(d$compartment, levels=c("Cortex","Medulla"))
    m <- lmer(pearson ~ group*compartment + (1|specimen), data=d)
    cat("\n==", pr, ": group x compartment interaction ==\n")
    print(round(summary(m)$coefficients, 4))
  }
}

cat("\n========== DONE. Outputs in ", OUTDIR, " ==========\n")
```
